# Complete Genome Sequence of Lactobacillus helveticus JCM 1004, an Aminopeptidase-Producing Lactic Acid Bacterium

**DOI:** 10.1128/MRA.00641-21

**Published:** 2021-08-19

**Authors:** Kana Morinaga, Hiroyuki Kusada, Miho Watanabe, Hideyuki Tamaki

**Affiliations:** a Bioproduction Research Institute, National Institute of Advanced Industrial Science and Technology, Tsukuba, Ibaraki, Japan; b Graduate School of Life and Environmental Sciences, University of Tsukuba, Tsukuba, Ibaraki, Japan; University of Rochester School of Medicine and Dentistry

## Abstract

We report the complete genome sequence of Lactobacillus helveticus JCM 1004, an aminopeptidase-producing lactic acid bacterium. The genome consists of a circular chromosome which comprises 2,261,280 bp, with a G+C content of 37.56%. The genome was predicted to harbor 13 rRNA genes, 64 tRNA genes, and 2,462 protein-coding sequences.

## ANNOUNCEMENT

Members of the genus *Lactobacillus* are Gram-positive, fermentative lactic acid bacteria belonging to the phylum *Firmicutes*. Among them, Lactobacillus helveticus strains have been widely known as industrial cheese starters (1, 2). The aminopeptidases produced by L. helveticus play a significant role in hydrolyzing milk proteins and releasing free amino acids involved in flavors during cheese ripening. L. helveticus JCM 1004 has also been reported to produce an aminopeptidase (3), though a gene encoding aminopeptidase has not been identified so far. Here, we performed a complete genome sequence analysis of L. helveticus JCM 1004 and identified putative aminopeptidase genes.

L. helveticus JCM 1004 (=LMG 1146 = NCDO 99 = NCIMB 700099) was obtained from the Japan Collection of Microorganisms (RIKEN BRC, Tsukuba, Japan). The strain was cultured on Gifu anaerobic medium (Nissui Pharmaceutical Co., Ltd., Tokyo, Japan) with a headspace gas of N_2_/CO_2_ (80:20, vol/vol) at 37°C for 48 h under anaerobic conditions. Full-grown cells were harvested by centrifugation and lysed with lysozyme, achromopeptidase, and proteinase K. Genomic DNA was extracted using the phenol-chloroform method as described previously (4). *De novo* sequencing using the HiSeq 2500 system (Illumina, San Diego, CA, USA) and the PacBio sequencing platform (Pacific Biosciences, Menlo Park, CA, USA) was conducted at Genewiz, Inc. (South Plainfield, NJ, USA) as described previously (5). The library was constructed using the VAHTS universal DNA library prep kit for Illumina (Vazyme Biotech, Nanjing, China). Illumina paired-end reads were generated, and the sequencing yielded a total of 9,065,976 reads. Default parameters were used for all bioinformatic software tools. Sequencing was carried out using a 2 × 150-bp paired-end configuration; image analysis and base calling were conducted using the HiSeq Control Software + OLB + GAPipeline v1.6 (Illumina) on the HiSeq instrument. Low-quality sequences and adaptors were removed using Cutadapt v1.9.1 (6). For PacBio sequencing, genomic DNA was sheared using a g-TUBE device (Covaris, Woburn, MA, USA) and then size selected and purified using magnetic beads. A SMRTbell library was constructed using the SMRTbell template prep kit v1.0 (Pacific Biosciences, Menlo Park, CA, USA) and then sequenced and analyzed using the PacBio RS II platform and single-molecule real-time (SMRT) sequencing technology (7). Quality control and adaptor trimming of the PacBio reads were performed using cutadapt v1.9.1; then, the reads were assembled using HGAP4/Falcon v0.3 of WGS-Assembler v8.2 (8). The PacBio sequencing yielded a total of 508,213 reads with an *N*_50_ value of 4,053 bp. The genome sequence was further corrected using the Illumina HiSeq and PacBio reads with Pilon v1.22 (9) and Quiver (10), respectively. The genome circularization was confirmed by PCR and Sanger sequencing across the ends of the chromosome. The resulting circular genome was evaluated for its completeness and contamination level using CheckM v1.0.7 (11). Protein-coding genes and prophage sequences were predicted using DFAST (12) and PHASTER (13), respectively. The amino acid sequences of putative aminopeptidases were further analyzed using the UniProt Web BLAST server (14).

The complete L. helveticus JCM 1004 genome comprises a circular 2,261,280-bp chromosome with an average coverage of 650×, a G+C content of 37.56%, 2,462 protein-coding sequences, 1 intact prophage, 13 rRNA genes, and 64 tRNA loci. Based on the CheckM analysis, the L. helveticus JCM 1004 genome was 99.03% complete with no contamination. The L. helveticus JCM 1004 genome encodes eight different aminopeptidase coding genes (Table 1). The complete genome sequence information of strain JCM 1004 will be useful for better understanding the physiological characteristics of the cheese starter and to further verify the enzymatic functions and production mechanisms of its aminopeptidases.

**TABLE 1:** Summary of putative aminopeptidases found in the L. helveticus JCM 1004 genome

Locus tag	Length (aa)^*a*^	Protein accession no.	Protein name	Source	E value	Identity (%)
LBHL_02250	437	P94870	Aminopeptidase E	Lactobacillus helveticus	3.4e−173	53.7
LBHL_02340	438	P94870	Aminopeptidase E	Lactobacillus helveticus	0.0	100
LBHL_03880	449	Q10744	Aminopeptidase C	Lactobacillus helveticus	0.0	98.4
LBHL_06230	504	M3XFH7	Aminopeptidase Q	Felis catus	1.3e−6	23.9
LBHL_14050	437	P94870	Aminopeptidase E	Lactobacillus helveticus	0.0	66.3
LBHL_18150	275	P19994	Methionine aminopeptidase 1	Bacillus subtilis strain 168	3.9e−69	44.7
LBHL_18880	360	O34924	Putative aminopeptidase YtoP	Bacillus subtilis strain 168	2.9e−47	33.1
LBHL_22300	844	Q10730	Aminopeptidase N	Lactobacillus helveticus	0.0	99.8

a aa, amino acids.

### Data availability.

The complete genome sequence and annotations of L. helveticus JCM 1004 have been deposited at DDBJ/EMBL/GenBank under accession number AP023028. The genome sequence also has been submitted to the SRA under BioSample accession number SAMD00215734 and BioProject accession number PRJDB9543. The raw sequence data for strain JCM 1004 were deposited under DRA accession numbers DRR287345 (Illumina) and DRR287346 (PacBio).
